# ORMDL3 contributes to the risk of atherosclerosis in Chinese Han population and mediates oxidized low-density lipoprotein-induced autophagy in endothelial cells

**DOI:** 10.1038/srep17194

**Published:** 2015-11-25

**Authors:** Xiaochun Ma, Rongfang Qiu, Jie Dang, Jiangxia Li, Qin Hu, Shan Shan, Qian Xin, Wenying Pan, Xianli Bian, Qianqian Yuan, Feng Long, Na Liu, Yan Li, Fei Gao, Chengwei Zou, Yaoqin Gong, Qiji Liu

**Affiliations:** 1 The Key Laboratory for Experimental Teratology of the Ministry of Education, Shandong University School of Medicine, Jinan, Shandong 250012, P.R. China; 2 Department of Medical Genetics, Shandong University School of Medicine, Jinan, Shandong 250012, P.R. China; 3 Department of Cardiac Surgery, Shandong Provincial Hospital affiliated to Shandong University, Jinan, Shandong 250021, P.R. China; 4 Department of Medical Genetics and Cell Biology, Ningxia Medical University, Yinchuan, Ningxia 750004, P.R. China; 5 The Key Laboratory of Cardiovascular Remodeling and Function Research, Chinese Ministry of Education and Chinese Ministry of Health, Qilu Hospital, Shandong University, Jinan, Shandong 250012, P.R. China; 6 Department of Obstetrics and Gynecology, Qilu Hospital, Shandong University, Jinan, Shandong 250012, P.R. China

## Abstract

ORMDL sphingolipid biosynthesis regulator 3 (ORMDL3) is a universally confirmed susceptibility gene for asthma and has recently emerged as a crucial modulator in lipid metabolism, inflammation and endoplasmic reticulum (ER) stress-the mechanisms also closely involved in atherosclerosis (AS). Here we first presented the evidence of two single nucleotide polymorphisms regulating ORMDL3 expression (rs7216389 and rs9303277) significantly associated with AS risk and the evidence of increased ORMDL3 expression in AS cases compared to controls, in Chinese Han population. Following the detection of its statistical correlation with AS, we further explored the functional relevance of ORMDL3 and hypothesized a potential role mediating autophagy as autophagy is activated upon modified lipid, inflammation and ER stress. Our results demonstrated that in endothelial cells oxidized low-density lipoprotein (ox-LDL) up-regulated ORMDL3 expression and knockdown of ORMDL3 alleviated not only ox-LDL-induced but also basal autophagy. BECN1 is essential for autophagy initiation and silencing of ORMDL3 suppressed ox-LDL-induced as well as basal BECN1 expression. In addition, deletion of ORMDL3 resulted in greater sensitivity to ox-LDL-induced cell death. Taken together, ORMDL3 might represent a causal gene mediating autophagy in endothelial cells in the pathogenesis of AS.

The complications of atherosclerosis (AS), such as coronary and cerebrovascular diseases, represent the most prevalent causes of morbidity and mortality worldwide^1^. A growing body of evidence has suggested the crucial role of chronic lipid-induced inflammation in mediating the initiation and progression of AS^2^. The genetic predisposition to AS has been recently recognized by the identification of susceptibility genes via genome-wide association studies (GWASs) and subsequent replication studies^3^. Since the first discovery of its association with asthma in 2007, ORMDL sphingolipid biosynthesis regulator 3 (ORMDL3) has been robustly confirmed in multiple ethnicities^4^,^5^. ORMDL3 has also been found associated with other immune-mediated chronic inflammatory diseases that include ulcerative colitis, type 1 diabetes and Crohn’s disease^6^,^7^,^8^. Situated in chromosome 17q21, a region in strong linkage disequilibrium (LD) and harboring well-defined single nucleotide polymorphisms (SNPs) regulating ORMDL3 expression^5^,^9^,^10^, the gene encodes a conserved endoplasmic reticulum (ER)-anchored protein with ubiquitous expression in adult and fetal tissues^11^. In recent functional investigations, ORMDL3 is emerging as a vital regulator in lipid homeostasis, inflammatory reaction, ER stress and unfolded protein response (UPR)–the mechanisms also closely involved in the pathogenesis of AS^12^,^13^,^14^,^15^. In addition, several studies proposed that asthma predicts a higher risk of coronary artery disease (CAD) and beyond that, experimental asthma accelerates AS in apolipoprotein E deficient (ApoE^−/−^) mice^16^,^17^. These results above-mentioned indicated the likelihood of chronic inflammatory disorders possessing genetic overlaps and the necessity of exploring whether ORMDL3 is genetically associated with AS.

Subsequent to the detection of statistical correlation of susceptibility genes with AS by genetic association studies, deciphering the major regulatory networks involved still remains a challenging issue. ORMDL3 disturbs ER-mediated calcium homeostasis and promotes ER stress and UPR^12^. In the context of asthma, aberrant ORMDL3 exacerbates airway inflammation and dysregulates ceramide metabolism^14^,^15^. Interestingly, potential factors that include ER stress, inflammation and dyslipidemia stimulate a survival mechanism–autophagy. Thus it is tempting to postulate the functional relevance of ORMDL3 in autophagy. Autophagy refers to an evolutionarily conserved catabolic process mediating lysosomal degradation of intracellular constitutes^18^. It involves the formation of double-membrane vesicles which engulf cytoplasmic components and refuse with lysosomes for turnover of aggregated proteins and damaged organelles^19^. Self-digestion of proteins and lipids can temporarily sustain stressed cells with an alternative source of energy, but can prove detrimental if overactive or prolonged^20^. And its role extends beyond cellular homeostasis maintenance to fine-tuning pathological processes such as tumorigenesis, AS, viral infection and neurodegeneration^21^,^22^,^23^.

It is becoming increasingly evident that autophagy participates centrally in the AS from the initial lesions to the end-stage thrombotic complications^24^,^25^. *In vitro* observations suggested the presence of autophagy in nearly all AS-relevant cell types including endothelial cells (ECs), macrophages and vascular smooth muscle cells (VSMCs). In AS successful autophagy could support cell survival and reverse cell injury and dysfunction. Especially, autophagy has been illuminated as a negative regulator of inflammatory reaction as well as oxidative stress^24^. Autophagy might also *in vivo* promote plaque stability, decelerate plague development and prevent plague rupture. Intensive studies for AS treatment focused on novel natural products and chemical reagents which triggered autophagy to retard atherogenesis^26^,^27^,^28^. The drug eluting stent (DES) with mammalian target of rapamycin (mTOR) inhibitors inducing autophagy has achieved widespread application in the management of CAD, which effectively lower restenosis rate after percutaneous coronary intervention (PCI)^29^. However, excessively activated autophagy might oppositely accelerate AS progression and clinical complications. Immoderate autophagy could not only lead to cell damage, dysfunction, apoptosis and even necrosis, but also promote pro-inflammatory responses^24^,^25^. Hence, exquisite control of autophagy to an appropriate extent might maximize its anti–atherosclerotic benefits and is potentially applied as a promising intervening method in treating AS. A novel finding by Wang *et al.* demonstrated that ORMDL1 and ORMDL3 were translocated out of the ER upon free cholesterol (FC) loading. FC loading-induced autophagy specifically degraded ORMDL1 to increase sphingomyelin biosynthesis which counters excess cholesterol^30^. How ORM proteins respond to other atherogenic stimuli is by far unknown and whether the family members could in turn mediate autophagy remains unclear.

Oxidized low-density lipoprotein (ox-LDL) is an established atherogenic inducer and some reported that simulation with ox-LDL to varying degrees initiated and perpetuated autophagy in ECs and macrophages^31^,^32^. Among all the atherosclerotic plague cells, ECs represent the earliest affected cell type and a crucial therapeutic target for AS treatment. Pharmaceutical strategies manipulating autophagy have manifested great potentials to control endothelial inflammation, dysfunction, aging and death^27^,^28^. However, the thorough understanding of genetic basis and major regulatory networks of autophagy is not sufficient in AS.

Here our results first confirmed a genetic association between ORMDL3 polymorphisms and AS risk in Chinese Han population. And our work extended beyond confirming susceptibility loci to clarifying underlying biological mechanisms. We provided a possible mechanism by which ox-LDL promotes autophagy via up-regulation of ORMDL3 in ECs.

## Results

### Clinical characteristics of study populations

The clinical characteristics of the included subjects (CAD cases and controls) from a northern Chinese cohort and a southern Chinese cohort were listed in Table 1. No significant difference was noted between the cases and controls in respect to age and gender. However, body mass index values, proportion of cigarette smoking, and triglyceride, total cholesterol and low-density lipoprotein cholesterol levels were notably higher whereas high-density lipoprotein cholesterol levels evidently lower in cases, compared to controls. The proportions of triple-vessel disease, acute coronary syndrome, PCI and coronary artery bypass grafting (CABG) of the case groups were also exhibited in Table 1.

### Genetic association study

The distributions of the 2 SNPs were in Hardy-Weinberg equilibrium (HWE) in cases and controls from both the northern and southern Chinese cohorts (*p* > 0.05).

In the northern Chinese cohort, the frequencies of rs7216389 T allele and rs9303277 C allele were higher in cases than in controls (rs7216389: 71.77% versus 67.07%; rs9303277: 68.50% versus 64.66%). rs7216389 and rs9303277 were significantly associated with AS after adjustment for age, sex, BMI, serum lipid levels and tobacco smoking (rs7216389: allelic *p* = 0.003; rs9303277: allelic *p* = 0.017). The subjects carrying the rs7216389 T allele and rs9303277 C allele showed an increased risk of AS (rs7216389: odds ratio [OR] 0.80, 95% confidence interval [CI] 0.69–0.93; rs9303277: OR 0.84, 95% CI 0.73–0.97) (Table 2).

Similarly in the southern Chinese population, higher frequencies of rs7216389 T allele and rs9303277 C allele were observed in cases than in controls (rs7216389: 75.22% versus 71.38%; rs9303277: 75.72% versus 72.28%) and the associations were replicated after adjustment for the covariates above-mentioned (rs7216389: allelic *p* = 0.009; rs9303277: allelic *p* = 0.022). Also the subjects carrying the rs7216389 T allele and rs9303277 C allele showed an increased risk of AS (rs7216389: OR 0.82, 95% CI 0.71–0.95; rs9303277: OR 0.84, 95% CI 0.72–0.97) (Table 2).

The 2 SNPs were in strong LD (r^2^ = 0.78 for the northern Chinese cohort; r^2^ = 0.89 for the southern Chinese cohort). With the sample size of each cohort and the alpha level set at 0.05, there was at least 80% power for detection of OR ≥1.2 for each SNP.

### Association of SNP alleles and gene expression of ORMDL3

**Figure 1:**
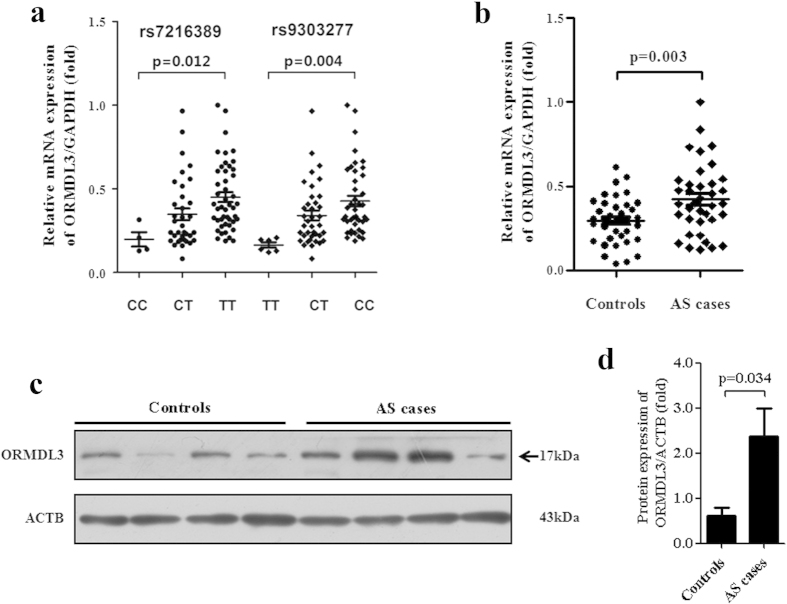
ORMDL3 expression among the included subjects. (**a**) Allele-specific mRNA levels of ORMDL3 by genotype of rs7216389 and rs9303277. (**b**) mRNA levels of ORMDL 3 between cases and controls. (**c–d**) Protein levels of ORMDL3 between cases and controls. The mRNA levels of ORMDL3 were normalized to that of GAPDH, and the protein levels normalized to that of ACTB. Each dot represents the mean value of 4 replicate samples for each subject.

The mRNA expression of ORMDL3 was significantly increased in cases carrying the risk alleles of rs7216389 and rs9303277 in comparison with homozygotes carriers of reference alleles in the northern Chinese cohort (rs7216389, *p* = 0.012; rs9303277, *p* = 0.004) (Fig. 1a).

ORMDL3 mRNA levels were significantly increased in cases compared to controls (*p* = 0.003) (Fig. 1b) and protein levels in accordance with the trend (*p* = 0.034) (Fig. 1c,d and supplementary Fig. S1).

### Up-regulation of ORMDL3 expression by ox-LDL in ECs

**Figure 2:**
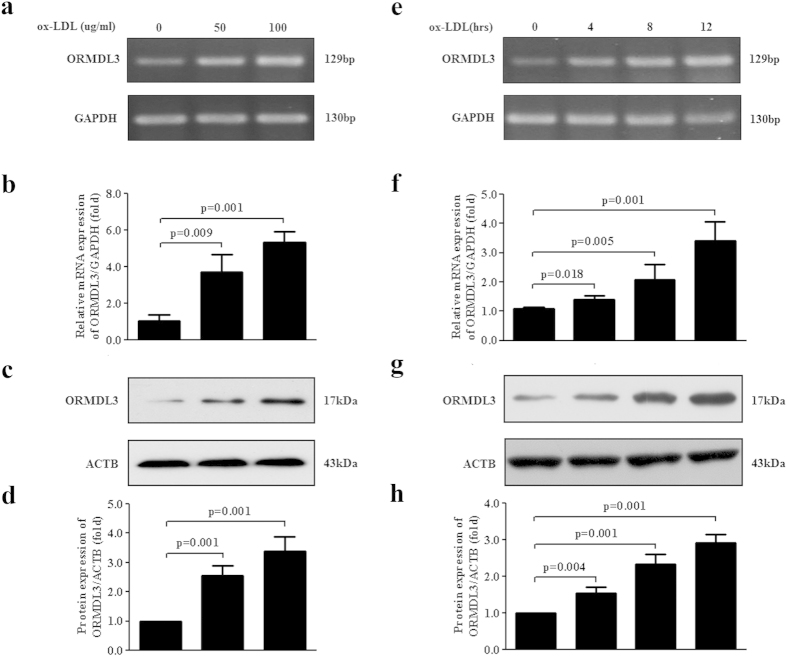
Ox-LDL up-regulates ORMDL3 expression in endothelial cells. (**a–d**) HUVECs were incubated with ox-LDL at various concentrations (0, 50 and 100 μg/ml) for 12 h. ORMDL3 mRNA and protein expression was measured by semi-quantitative PCR, qRT-PCR and immunoblot. (**e–h**) HUVECs were stimulated with 100 μg/ml ox-LDL for the indicated durations (0, 4, 8 and 12 h). ORMDL3 mRNA and protein levels were examined by semi-quantitative PCR, qRT-PCR and immunoblot. All the results shown above are the mean ± SD values (n = 3). Abbreviations: ox-LDL, oxidized low-density lipoprotein.

To further determine the effects of ox-LDL on ORMDL3 expression and whether the effect is *in vitro* dose-dependent and time-dependent, HUVECs, a widely applied model for endothelial cells, were stimulated with ox-LDL at various concentrations (0, 50 and 100 μg/ml) for different durations (0, 4, 8 and 12 h) and mRNA and protein levels were measured by semi-quantitative PCR, qRT-PCR and immunoblot. As shown in Fig. 2a,b, ox-LDL incubation led to a progressive increase in ORMDL3 mRNA in response to 0–100 μg/ml ox-LDL for 12 h. Consistent with the mRNA result, ox-LDL increased ORMDL3 protein content in a dose-dependent manner (Fig. 2c,d). Additionally, treating HUVECs with 100 μg/ml ox-LDL caused a time-dependent increase in the expression of ORMDL3 mRNA and protein (Fig. 2e–h). Similarly in human aortic endothelial cells (HAECs), another common model for endothelial cells, incubation with various concentrations of ox-LDL for different durations *in vitro* dose- and time-dependently up-regulated ORMDL3 protein levels, indicating a consistent responsiveness of different endothelial cell types to ox-LDL stimulation (Supplementary Fig. S2).

### Ox-LDL up-regulates autophagy in ECs

**Figure 3:**
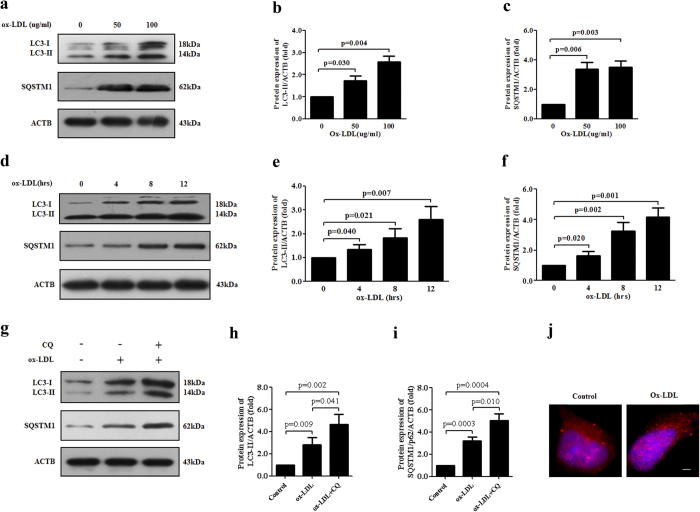
Autophagy is induced by ox-LDL in endothelial cells. (**a–c**) Following incubation with ox-LDL at various concentrations (0, 50 and 100 μg/ml) for 12 h in HUVECs, representative immunoblots of autophagy markers LC3 and SQSTM1/p62 were performed. (**d–f**) Immunoblot detection of protein levels of LC3 and SQSTM1/p62 in HUVECs treated with 100 μg/ml ox-LDL for different durations (0, 4, 8 and 12 h). (**g–i**) HUVECs were treated with ox-LDL alone or a combination of ox-LDL (100 μg/ml) and CQ (50 uM) for 12 h. The protein levels of LC3 and SQSTM1/p62 were monitored by immunoblot. (**j**) After stimulation with 100 μg/ml ox-LDL for 12 h, HUVECs were observed via immunofluorescence assay under fluorescence microscopy to evaluate the LC3 subcellular localization (red). DAPI was used to visualize nuclei (blue). All the results shown above are the mean ± SD values (n = 3). Scale bar: 10 μm. Abbreviations: ox-LDL, oxidized low-density lipoprotein; CQ, chloroquine.

Ox-LDL is a well-documented AS risk factor and a potent autophagy inducer. A finding by Muller *et al.* showed that ox-LDL triggers a time-dependent increase in LC3-II in ECs^31^,^32^. We next explored the full details of ox-LDL-induced autophagy in HUVECs. Similarly as aforementioned, HUVECs were exposed to increasing doses of ox-LDL (0, 50 and 100 μg/ml) for various durations (0, 4, 8 and 12 h). Autophagosome formation was detected using immunoblot assay for the lipidated MAP1LC3/LC3, a marker for autophagy. Following exposure to ox-LDL, LC3-II levels increased in a dose-dependent and time-dependent manner. And ox-LDL-stimulated HUVECs displayed increased amounts of autophagosome cargo SQSTM1/p62, a result in accordance with the previous findings by Peng *et al.* in ECs^29^ and by Choi *et al.* in macrophages^33^ (Fig. 3a–f). Increased levels of LC3-II could be attributable to either enhanced autophagic activity or impaired LC3 degradation. To monitor autophagic flux for the analysis of autophagy, the lysosomal inhibitor CQ which inhibits the fusion between autophagosomes and lysosomes was used in combination with ox-LDL. Co-stimulation of CQ plus ox-LDL in HUVECs resulted in further accumulation of LC3-II and SQSTM1/p62 (Fig. 3g–i). To further validate these results, we used fluorescence microscopy to observe the effects of ox-LDL on autophagy in HUVECs. Ox-LDL-incubated HUVECs exhibited the redistribution of LC3 to form puncta whereas in untreated cells LC3 was diffusely distributed in the cytoplasm (Fig. 3j). To investigate whether ox-LDL is also an efficient autophagy inducer for HAECs, autophagy markers LC3-II and SQSTM1/p62 were determined in HAECs treated with increasing doses of ox-LDL for various durations as abovementioned. As well, stimulation with ox-LDL resulted in pronounced autophagic activity, as demonstrated by an increase in LC3-II conversion and SQSTM1/p62 accumulation (Supplementary Fig. S3).

### Knockdown of ORMDL3 suppresses ox-LDL-induced autophagy in ECs

**Figure 4:**
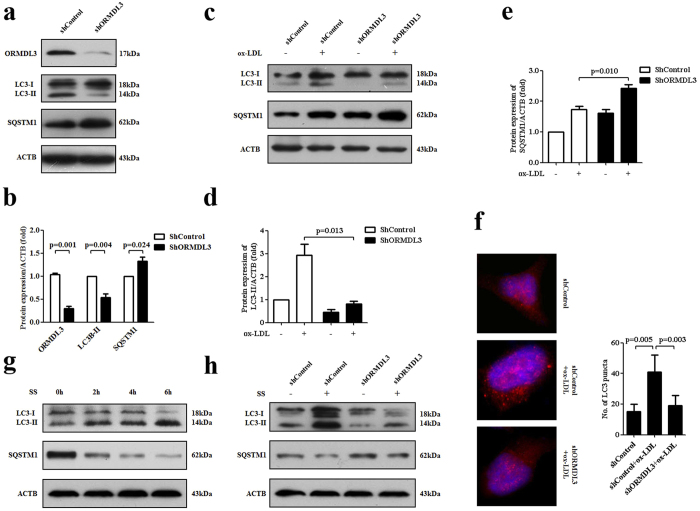
Knockdown of ORMDL3 suppresses basal and ox-LDL-induced autophagy. (**a–b**) At basal condition, in HUVECs stably transfected with ORMDL3 shRNA and control shRNA, transfection efficiency and protein levels of LC3 and SQSTM1/p62 were evaluated by immunoblot. (**c–e**) Immunoblot detection of protein levels of LC3 and SQSTM1/p62 in ShORMDL3 and shControl cells treated with 100 μg/ml ox-LDL or PBS for 12 h. (**f**) After stimulation with 100 μg/ml ox-LDL or PBS for 12 h, ShORMDL3 and shControl cells were observed by fluorescence microscopy to evaluate the LC3 pattern (red). DAPI was used to visualize nuclei (blue). The numbers of LC3 puncta per cell were counted (n = 10). (**g**) HUVECs were cultured in serum-free DMEM for various durations (0, 2, 4 and 6 h) and protein levels of LC3 and SQSTM1/p62 were evaluated by immunoblot. (**h**) Immunoblot detection of protein levels of LC3 and SQSTM1/p62 in ShORMDL3 and shControl cells challenged with serum starvation or not for 6 h. All the results shown above are the mean ± SD values (n = 3). Scale bar: 10 μm. Abbreviations: ox-LDL, oxidized low-density lipoprotein; SS, serum starvation.

We subsequently questioned whether ORMDL3 was essentially required for basal and ox-LDL-induced autophagy in ECs. As shown, knockdown of ORMDL3 decreased LC3-II level and consistently increased SQSTM1/p62 level in HUVECs under basal condition, suggesting that ORMDL3 knockdown suppressed basal autophagy in part (Fig. 4a,b). We further explore the effect of knockdown of ORMDL3 on ox-LDL-induced autophagy. As expected, knockdown of ORMDL3 partially neutralized the conversion to LC3-II and augmented the SQSTM1/p62 accumulation by ox-LDL (Fig. 4c–e). Although under basal condition it was difficult to observe LC3-II puncta, knockdown of ORMDL3 led to less LC3-II puncta formation by ox-LDL (Fig. 4f). Besides, we demonstrated that serum starvation challenge to HUVECs from 0 h to 6 h dramatically enhanced autophagy in HUVECs, which was also alleviated by knockdown of ORMDL3 (Fig. 4g,h).

### ORMDL3 might regulate autophagy via BECN1

**Figure 5:**
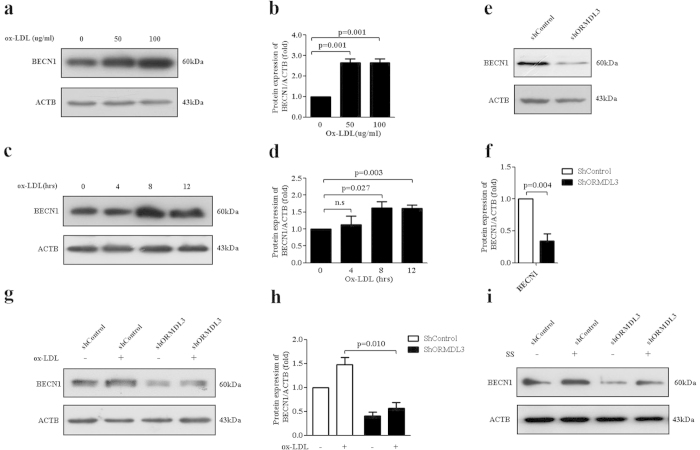
ORMDL3 might regulate autophagy via BECN1. (**a–b**) BECN1 protein levels were measured in HUVECs treated with ox-LDL at various concentrations (0, 50 and 100 μg/ml) for 12 h by immunoblot. (**c–d**) Immunoblot detection of protein levels of BECN1 in HUVECs treated with 100 μg/ml ox-LDL for different durations (0, 4, 8 and 12 h). (**e–f**) At basal condition, in ShORMDL3 and shControl HUVECs, BECN1 protein levels were evaluated by immunoblot. (**g–h**) Immunoblot detection of protein levels of BECN1 in ShORMDL3 and shControl cells treated with 100 μg/ml ox-LDL or PBS for 12 h. (**i**) Immunoblot detection of protein levels of BECN1 in ShORMDL3 and shControl cells challenged with serum starvation or not for 6 h. All the results shown above are the mean ± SD values (n = 3). Abbreviations: ox-LDL, oxidized low-density lipoprotein; SS, serum starvation.

BECN1 has long been recognized as crucial autophagy marker and it is required for ER-stress induced autophagy^34^. BECN1 is induced upon ox-LDL stimulation by time course experiments^31^,^32^ in endothelial cells. Here we investigated whether BECN1 was involved in ORMDL3-regulated autophagy in HUVECs. We noticed that HUVECs treated with increasing concentrations of ox-LDL for various durations displayed enhanced protein expression of BECN1 (Fig. 5a–d). And knockdown of ORMDL3 alleviated not only basal (Fig. 5e,f) but also ox-LDL-induced (Fig. 5g,f) and serum starvation-induced BECN1 levels (Fig. 5i). Hence, it is hypothesized that ORMDL3 regulates autophagy via BECN1.

To explore the potential regulatory factors upstream of BECN1 in ORMDL3-mediated autophagy, it was investigated whether ORMDL3 might exert effect on mammalian target of rapamycin (mTOR) pathway-one major pathway involved in starvation-induced and ox-LDL-induced autophagy. However, no alteration in phosphorylated mTOR level was noted due to ORMDL3 knockdown, as was the level of phosphorylated AMP-activated protein kinase (AMPK) (Supplementary Fig. S4).

Besides, for the purpose of investigating whether ORMDL3 is potentially implicated in ox-LDL-strengthened inflammation in HUVECs, the mRNA levels of several well-known inflammatory factors including tumor necrosis factor (TNF), IL-6 and IL-17A in ox-LDL-stimulated shORMDL3 and shControl cells were determined by qRT-PCR. Ox-LDL expectedly augmented the mRNA expression of TNF, IL-6 and IL-17A in HUVECs. And ORMDL3 knockdown neutralized the ox-LDL-induced expression of TNF and IL-17A but not IL-6 (Supplementary Fig. S5).

### Knockdown of ORMDL3 promotes ox-LDL-induced apoptosis in ECs

**Figure 6:**
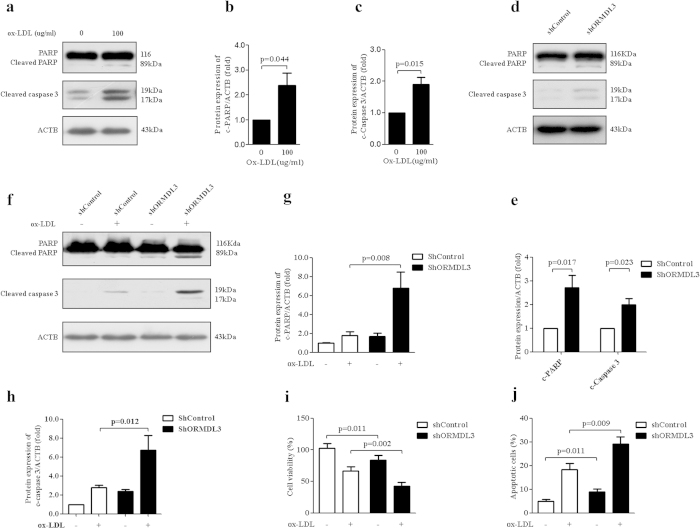
Knockdown of ORMDL3 promotes ox-LDL-induced apoptosis in endothelial cells. (**a–c**) Immunoblot detection of protein levels of cleaved PARP and caspase 3 in HUVECs treated with 100 μg/ml ox-LDL for 12 h. (**d–e**) At basal condition, in ShORMDL3 and shControl HUVECs, protein levels of cleaved PARP and caspase 3 were evaluated by immunoblot. (**f–h**) Immunoblot detection of protein levels of cleaved PARP and caspase 3 in ShORMDL3 and shControl cells treated with 100 μg/ml ox-LDL or PBS for 12 h. (**i**) Cell viability measured using CCK-8 assay in ShORMDL3 and shControl cells stimulated with 100 μg/ml ox-LDL or PBS for 12 h. (**j**) Apoptosis rates determined using Annexin V-PI staining by flow cytometry in ShORMDL3 and shControl cells incubated with 100 μg/ml ox-LDL or PBS for 12 h. Annexin V-PI staining shows the percentage of apoptotic cells. All the results shown above are the mean ± SD values (n = 3). Abbreviations: ox-LDL, oxidized low-density lipoprotein; c-PARP, cleaved PARP; c-caspase 3, cleaved caspase 3.

Ox-LDL potently triggers apoptotic process in ECs^31^,^32^. Our question was whether knockdown of ORMDL3 would affect ox-LDL-induced apoptosis. Our findings demonstrated that stimulation with 100 μg/ml ox-LDL for 12 h led to enhanced protein levels of cleaved caspase 3 and PARP (Fig. 6a–c). While under basal condition knockdown of ORMDL3 promoted caspase 3 and PARP cleavage (Fig. 6d,e); with ox-LDL stimulation knockdown of ORMDL3 markedly exacerbated apoptosis, demonstrated by augmented caspase 3 and PARP cleavage (Fig. 6f–h), cell viability impairment and apoptotic cell rates (Fig. 6i,j). It implied that ORMDL3 might represent a protective mechanism against ox-LDL toxicity.

## Discussion

AS turns out to be a complex and multifactorial disorder in which genetic and environmental factors contribute interactively to disease pathogenesis^35^. While the major risk factors have long been recognized, the detailed interpretation of genetic mechanisms is just in the preliminary phase. We undertook the present study to investigate the association of ORMDL3 polymorphisms and AS in Chinese Han population and the functional relevance of ORMDL3 in AS. Based on the current results, we first identified two SNPs situated in Chr. 17q21 containing ORMDL3, rs7216389 and rs9303277, significantly associated with AS risk in two Chinese Han populations. In addition, we showed the elevated mRNA and protein levels of ORMDL3 in peripheral leukocytes from AS cases than controls. As well, the risk alleles for the two SNPs were linked to increased ORMDL3 mRNA levels in leukocytes from patients. The observed association was repeatedly confirmed in two populations, which added credibility to our findings. However, there is a possibility that rs7216389 and rs9303277 is in strong LD with the true disease-associated loci, given the LD status within 17q21 and the fact that the two SNPs maps within GSDMB and IKZF3 instead of ORMDL3. Hence, the detailed mechanisms of the SNPs affecting mRNA abundance, such as mRNA splicing, transcription factor binding and microRNA targeting, remains to be further clarified. Besides, additional variants within 17q21 are awaiting verification to target disease-associated loci for AS, and the association has also to be robustly replicated in other ethnicities.

Despite remarkable achievements in recent years, the utmost importance of autophagy is still largely underestimated in AS^25^. Clarifying how genetic factors influence autophagy is evidently relevant and one great challenge remains the identification of autophagy-related genes to deepen our understanding of major pathways involved. Following association study, we further dug into mechanistic investigation for ORMDL3 in AS. Here we uncovered a plausible interpretation for autophagy in AS that entails up-regulation of ORMDL3 by ox-LDL. In ox-LDL-stimulated HUVECs and HAECs, ORMDL3 induction was time- and dose-dependent at both mRNA and protein level. The observation enriched the knowledge of inducing factors for ORMDL3 apart from Th2 cytokines interleukin (IL)-4 and IL-13^36^,^37^. We next demonstrated that ox-LDL promotes autophagy activity in HUVECs and HAECs in which a time- and concentration-dependent conversion of LC3I to LC3II occurs. And ox-LDL incubation led to accumulation of SQSTM1/p62 and this was not discordant with the findings by other groups in ECs and macrophages, though SQSTM1/p62 is generally degraded when autophagy strengthens due to common stressors such as nutrition deprivation^29^,^33^. We then noticed that ox-LDL-triggered autophagy is in part alleviated by shRNA-mediated silencing of ORMDL3. Not specific to ox-LDL induction, knockdown of ORMDL3 also suppressed basal and serum starvation-induced autophagy, supporting a fundamental role of ORMDL3 in autophagy through an unidentified mechanism. Upon autophagy initiation, BECN1 is released from BECN1-BCL2 complex for inhibition freeing and it has proved to be a vital autophagy gatekeeper^38^. We subsequently showed that ox-LDL up-regulates BECN1 protein level and knockdown of ORMDL3 reverts this effect and also suppresses basal and serum starvation-induced BECN1 level. Incorporating our results with the previous finding that BECN1 regulates ER stress-induced autophagy^34^, it is reasonable to propose that ORMDL3 regulates autophagy via BECN1. However, the detailed mechanism by which ORMDL3 mediates BECN1 expression is undefined and it remains unknown whether ORMDL3 could also disturb the BECN1-BCL2 interaction. Furthermore, mTOR pathway-the most recognized and studied autophagy regulator-is proposed not to be relevant in ORMDL3-regulated autophagy based on our results, an assumption that needs further in-depth analysis. ER stress and UPR are among well-defined autophagy inducers by a mechanism in which 78-kDa glucose-regulated protein (GRP78/Bip) is required^34^,^39^. Deletion of ORMDL3 *in vitro* decreases GRP78/Bip level^12^. Thus it is inevitable to speculate an ORMDL3-ER stress/UPR-BECN1-autophagy regulatory pathway particularly relevant in ORMDL3-mediated autophagy. Previous work has illuminated *in vivo and in vitro* that ORMDL3 facilitates ER stress and UPR mainly through the activation of Activating Transcription Factor 6 (ATF6) pathway^12^,^14^. However, we are just in the preliminary phase to verify this assumption and to confirm which specific ER stress pathway is indeed involved. We finally showed a defensive effect of ORMDL3 against ox-LDL-induced apoptosis. Consistently, prior work found that double knockout of two yeast homologs resulted in decreased growth rate and higher sensitivity to DTT and tunicamycin, two well-known ER stress inducers. The phenotypic defects could be rescued by functional complementation with human homologs^11^. These results indicated a protective role of ORM members against environmental threats. Strikingly, Muller *et al.* proposed that BECN1 (actually autophagic process) is independent of the pro-apoptotic arm of ox-LDL^28^,^29^. Thus it has yet to be investigated whether this beneficial effect of ORMDL3 is mediated through autophagy regulation.

Autophagy is stimulated by various atherogenic stimuli that include oxidized lipids, ER stress activation, inflammation, hypoxia and metabolic stress^25^. Its alterations are verifiably observed in model cell lines, AS-prone model mice and human coronary and carotid plaques^24^,^40^. The role of autophagy in AS remains poorly understood, with evidence suggesting both protective and detrimental effects. Most likely in AS, autophagy defends against cellular stress as it is well established as a life-sustaining mechanism. By degrading damaged proteins and organelles and disposing aggregated lipids, autophagy contributes to cellular recovery in adverse environments, in particular oxidative stress and inflammation^24^,^41^. What deserves close attention is that specific natural and synthetic compounds could utilize a protective autophagy for stress repairing against AS. Statins, anti-cholesterol drugs widely administrated for treating AS, are known to induce cultured VSMC apoptosis, which is partially rescued by autophagy inducer 7-ketocholesterol^42^. Verapamil, a well-known calcium channel blockers for hypertension therapy, confers anti-proliferative benefits in VSMC to control neointima formation via up-regulation of autophagy^43^. Resveratrol, a polyphenolic compound mainly extracted from the skin of grapes, could increase autophagy to restore endothelial homeostasis following tumor necrosis factor stimulation^27^. Berberine, an alkaloid extracted from *Coptis*, alleviates ox-LDL induced inflammatory factors in macrophages by up-regulation of autophagy^26^. In addition, Delphinidin-3-glucoside and curcumin protects ECs against ox-LDL induced injury and oxidative stress damage, both via induction of autophagy^28^,^44^. Interestingly, a synthetic chemical 3BDO as mTOR activator inhibits ox-LDL-induced autophagy and apoptosis in ECs and *in vivo* reverses AS development in ApoE^−/−^ mice^29^. Besides, genetic engineering mouse models of AS have concluded the athero-protective role of autophagy *in vivo*. Knockdown of Atg5, an essential autophagy gene, augments AS progression through promoting apoptosis and oxidative stress, impairing cholesterol efflux from macrophages, facilitating formation of cholesterol crystals and lesional necrosis, inducing pro-atherogenic inflammasome activation and worsening lesional efferocytosis^45^,^46^. Consistently, silencing Wip1, an mTOR dependent autophagy inhibitor, positively regulates cholesterol efflux in macrophages and reduces AS development^47^. In addition, autophagy is also impaired in aging ECs and associated with EC dysfunction in response to ox-LDL treatment^48^. In contrast to beneficial autophagy, aberrantly activated autophagy could trigger autophagic death of VSMCs and ECs, due to excessive elimination of cytosol and organelles. It might reduce collagen contents and render the plague vulnerable to rupture and thrombosis. Thus autophagy might affect plague stability and eventually lead to clinical thrombotic events^25^,^48^,^49^. The Janus face of autophagy in AS and beyond that, the technical limitations for detection and lack of accurate biomarkers, makes it complicated to be manipulated as targets for preventive and therapeutic interventions^41^. And further work is still necessary to decipher the critical regulatory networks of this phenomenon involved in AS.

ORMDL3 reinforces ER stress, a potent intercellular inflammatory activator. Recent work has unveiled the relevance of ORMDL3 in inflammation; however, most findings were confined to airway inflammation, remodeling and hypersensitivity in the context of asthma. Transfection of OMRDL3 *in vitro* induces the expression of metalloproteases, chemokines, oligoadenylate synthetases (OAS), ATF6 and SERCA2b in human bronchial epithelial cells^12^. Moreover, ORMDL3 *in vivo* exacerbates airway inflammation, remodeling and responsiveness along with increased levels of ATF6 and SERCA2b in ORMDL3 transgenic mice^14^. In allergic eosinophilic disorders such as asthma, ORMDL3 could also mediate eosinophil trafficking, recruitment and degranulation via integrin and CD48^50^. Additionally, with its highly conserved family members, ORMDL3 participates in sphingolipid regulation as the vital rate limiting enzyme at the first step of sphingomyelin synthesis and ORMDL3 aberrance dysregulates ceramide homeostasis, implying that ORMDL3 might contribute to abnormal lipid metabolism in AS^15^,^51^. Besides, IL-17 could accentuate vascular and systemic inflammation and modulate plaque stability in experimental AS^52^. Polymorphisms mapping in 17q21 strikingly elevated ORMDL3 level and interleukin-17 (IL-17) secretion in cord blood, implying ORMDL3 might correlate with Th17 pathway^53^. We found that ORMDL3 knockdown neutralized the ox-LDL-induced expression of TNF and IL-17A but not IL-6. These observations shed a light on a potential mechanism that entails a role of ORMDL3 in regulation of inflammation in AS. As well, macrophage inflammation and apoptosis are the key cellular events in AS plaques conversion and lesion macrophages derived from peripheral monocytes are one of the major components in advanced plaques^54^. We noted the levels of ORMDL3 significantly increased in peripheral blood leukocytes in AS cases compared with controls, and also an allele-specific expression pattern. These observations were indicative of a role of ORMDL3 in macrophages and it deserves intensive investigation in near future.

Taken together, the unsolved issues regarding ORMDL3 include in AS pathogenesis: how ORMDL3 regulates autophagy in full detail and whether it is *in vivo* involved in plague formation and progression; whether autophagy impacts ORMDL3 in reverse; and whether ORMDL3 is implicated in other vital regulatory networks that include inflammation, oxidative stress, ER stress and lipid metabolism in various cell types.

## Methods

### Study populations

From 2010 to 2014, a total of 1,829 AS (coronary artery disease, [CAD]) cases and 1,806 unrelated healthy controls matched to cases by age, gender, ethnicity and geographic region, were consecutively enrolled. 898 cases (603 males mean age 60.01 ± 9.87 years) and 915 controls (606 males, mean age 60.26 ± 7.34 years) were recruited from Qilu Hospital in Shandong Province, a province in northern China. 931 cases (569 males mean age 63.89 ± 10.81 years) and 891 controls (553 males, mean age 63.51 ± 10.25 years) were recruited from Tongji Hospital in Hubei Province in southern China. All the subjects underwent comprehensive medical evaluation including echocardiography, carotid ultrasonography and coronary angiography at the time of enrollment. The diagnosis of CAD was established by at least 2 experienced cardiologists. Individuals with more than 70% coronary stenosis in at least one main vessel confirmed by coronary angiography or computed tomography angiography (CTA) were designated as eligible, as were those who experienced a history of myocardial infarction and/or underwent percutaneous coronary intervention (PCI) or coronary artery bypass graft (CABG). Subjects with peripheral vascular disease, chronic kidney, pulmonary heart disease, hepatic disease and rheumatic heart disease were excluded. Ethics approval for this study was obtained from the ethics review committee for human studies of School of Medicine, Shandong University, and patients provided their informed consent.

### Single nucleotide polymorphism selection and genotyping

Genomic DNA was extracted from peripheral blood leukocytes by a standard phenol-chloroform method. We analyzed the LD situation of chromosome 17q21 region containing ORMDL3 gene (chromosome 17: 35170k–35350k) using the HapMap HCB data (HapMap3 genome browser release #3) by Haploview 4.2. Two SNPs, rs7216389 and rs9303277, were selected with minor allele frequencies ≥5% and r^2^ > 0.8 in HCB population. The SNPs were genotyped by the Taqman method with assay-on demand probes and primers (C_29062108_10 for rs7216389, C_9272050_20 for rs9303277). We used the Applied Biosystems 7500 Real-time PCR system and SDS 1.4 Automation controller software for genotyping. Genotyping accuracy in the samples was confirmed by direct sequencing of PCR products for 5% randomly chosen samples.

### RNA isolation and quantitative real-time PCR

Total RNA was extracted using TRIZOL reagent method following the manufacturer’s protocol (Invitrogen, Carlsbad, CA, USA). Reverse transcription PCR was performed using the MMLV Reverse Transcriptase (Thermo Scientific, Rockford, IL, USA). Relative mRNA levels were determined using SYBR Green Real Time PCR kit (TIANGEN, Beijing, China) in the Roche LightCycler® 480 System with GAPDH as the internal control. The comparative threshold cycle method was used for quantification with 4 wells duplicated. Especially, allelic-specific ORMDL3 mRNA expression (89 cases) and the difference in ORMDL3 mRNA levels between cases and controls (37 cases versus 37 controls) were quantitatively analyzed for the subjects from the northern Chinese cohort.

The primers used for real-time PCR are listed as follows:

forward, 5′-GACCCTCACCAACCTCATTCAC-3′,

reverse, 5′-CCATAATCCATCTGCTCCCAGTG-3′ for human ORMDL3 gene;

forward, 5′-CCAGGTGGTCTCCTCTGACTT-3′,

reverse, 5′-GTTGCTGTAGCCAAATTCGTTGT-3′ for human GAPDH gene;

forward, 5′-CCTCTCTCTAATCAGCCCTCTG -3′,

reverse, 5′-GAGGACCTGGGAGTAGATGAG -3′ for human TNF gene;

forward, 5′-ACTCACCTCTTCAGAACGAATTG-3′,

reverse, 5′-CCATCTTTGGAAGGTTCAGGTTG-3′ for human IL-6 gene;

forward, 5′-TCCCACGAAATCCAGGATGC-3′,

reverse, 5′-GGATGTTCAGGTTGACCATCAC-3′ for human IL-17A gene.

### Antibodies and reagents

Primary antibodies against BECN1, PARP, cleaved Caspase 3, phosphorylated (p) mTOR, total mTOR, p-AMPK and total AMPK were purchased from Cell Signaling Technology (CST, Danvers, MA, USA), antibodies against SQSTM1/p62, MAP1LC3B and ORMDL3 from Abcam (Hong Kong, China), and antibody against ACTB from Santa Cruz Biotechnology (Santa Cruz, Dallas, TX, USA). Ox-LDL was purchased from Yiyuan Biotechnologies (Guangzhou, China) and chloroquine (CQ) and puromycin from sigma (St Louis, MO, USA).

### Cell culture and treatment

Human umbilical vein endothelial cells (HUVECs), obtained from American Type Culture Collection (ATCC, VA, USA), were maintained in Dulbecco’s modified Eagle’s medium (DMEM) (Gibco Laboratories, Grand Island, NY) supplemented with 10% fetal bovine serum (FBS), endothelial cell growth supplement (ECGS, ScienCell) and 100 U/ml penicillin-streptomycin at 37 °C in a humidified incubator containing 5% CO_2_. Human aortic endothelial cells (HAECs) were purchased from ATCC and cultured in endothelial culture medium (ECM, ScienCell, CA, USA) supplemented with 5% FBS, ECGS and 100 U/ml penicillin-streptomycin at 37 °C with 5% CO_2_.

HUVECs and HAECs were stimulated with various concentrations of ox-LDL (0, 50 and 100 μg/ml) for different durations (0, 4, 8 and 12 h), or treated with a combination of ox-LDL (100 μg/ml) and CQ (50 uM) for 12 h. In control groups, cells were treated with phosphate buffered saline (PBS). For serum starvation-induced autophagy, HUVECs were cultured in serum-free DMEM for various durations (0, 2, 4 and 6 h).

### Cell transfection

All retroviruses were produced by transfecting the pGIPZ constructs expressing small hairpin RNAs (shRNA)–shORMDL3 and shControl–into the HEK293T packaging cell line. For transfection, HUVECs were plated at a density of 2.5 × 10^4^/35 mm plate and cultured for 24 hours before transfection. Then medium containing packaged viruses was added and 48 hours later was replaced by fresh medium supplemented with 2 μg/ml puromycin for selection which lasts 2 weeks to establish stably transfected cell lines.

### Evaluation of cell viability and apoptosis

For cell viability evaluation, HUVECs were seeded in the 96-well plates at the density of 5000 cells/well 12 h ahead of time and then incubated with 100 μg/ml ox-LDL. At 12 h following stimulation, cell counting kit (CCK-8) solution (BestBio, Shanghai, China) (10 μl/well) was added to each well and the plates were incubated at 37 °C for 2 h. Measurements of cell viability were determined by absorbance at the wavelength of 450 nm and showed as the percentages in proportion to control groups.

For apoptosis evaluation, HUVECs were harvested and washed twice with cold PBS before re-suspended and stained with 5 μl Annexin V and Propidium Iodide (PI) for 15 min in accordance with the manufacturer’s instructions (BD Biosciences, San Diego, CA, USA). Then apoptosis was analyzed using FACScan flow cytometer with Annexin V and PI emissions detected. The percentage of Annexin V^+^/PI^+^ and annexin V^+^/PI^−^ cells was determined and compared with controls. Besides, apoptosis-related proteins, cleaved caspase3 and PARP, were evaluated by immunoblot.

### Immunoblot

Total cell lysates were collected in lysis buffer (Bioteke, Beijing, China) supplemented with 1% protease inhibitor and protein concentrations were measured using the BCA method (Thermo). 30–50 μg of whole cell lysates were resolved by SDS-PAGE using 8% or 12% gels and transferred onto PVDF membranes (Roche; Roche Diagnostics, Basel, Switzerland). Then the membranes were blocked with 5% nonfat milk in Tris-buffered saline containing 0.2% Tween 20 for 1 hour at room temperature. For immunodetection, the membranes were incubated with 1:200–1:5000 diluted primary antibodies overnight at 4 °C and then secondary antibodies conjugated with horseradish peroxidase for 1 h at room temperature. The signals were visualized by ECL blotting detection reagents (Thermo) and exposed to X-ray films which were scanned and quantitatively analyzed by Quantity One (Bio-Rad, California, USA). ACTB was used as corresponding loading control.

### Cell staining for immunofluorescence microscopy

Treated HUVECs were fixed by 4% paraformaldehyde for 30 min and blocked with 10% normal goat serum for 1 h at room temperature. Then cells were incubated with anti-LC3 antibody (1:50) overnight at 4 °C, then probed with corresponding secondary antibody for 1 h at 37 °C and evaluated by laser-scanning confocal microscopy. In control groups, cells were incubated with normal IgG.

### Statistical analysis

All results were shown as mean ± SD or no. (%). Clinical characteristics were compared using the Student’s t test or Fisher’s exact test by SPSS for Windows v13.0 (SPSS Inc., Chicago, IL, USA). SNPs were tested using chi-square test for deviation from HWE by SPSS. Genetic association analysis involved the use of PLINK 1.07 (http://pngu.mgh.harvard.edu/~purcell/plink/) and odds ratios (ORs) and 95% confidence intervals (CIs) for the minor alleles were calculated after adjusting for covariates (including age, sex, BMI, serum lipid levels and tobacco smoking) with unconditional logistic regression by SPSS. When making multiple statistical comparisons, Bonferroni correction was applied to avoid an inflated Type I error rate. Pairwise LD was measured by use of Haploview 4.2. Power calculation was performed using the CaTS power calculator to evaluate the Type II error (http://csg.sph.umich.edu/abecasis/CaTS/). One-way analysis of variance (ANOVA) with Student-Newmann-Keuls test for comparison between three groups and Student’s t test for comparison between two groups were performed for functional studies by SPSS. Images were processed by use of Graphpad Prism 5 (GraphPad Software, La Jolla, CA, USA). A *p* value of less than 0.05 was considered statistically significant.

## Additional Information

**How to cite this article**: Ma, X. *et al.* ORMDL3 contributes to the risk of atherosclerosis in Chinese Han population and mediates oxidized low-density lipoprotein-induced autophagy in endothelial cells. *Sci. Rep.* **5**, 17194; doi: 10.1038/srep17194 (2015).

## Supplementary Material

Supplementary Information

## Figures and Tables

**Table 1:** General characteristics of the study population.

Variables	The northern Chinese population			The southern Chinese population		
	Cases (n = 898)	Controls (n = 915)	*p*values	Cases (n = 931)	Controls (n = 891)	*p*values
Age (years)	60.01 ± 9.87k	60.26 ± 7.34	ns	63.89 ± 10.81	63.51 ± 10.25	ns
Male, no. (%)	603 (67.15)	606 (66.23)	ns	569 (61.12)	553 (62.07)	ns
BMI (kg/m^2^)	25.98 ± 3.41	23.70 ± 3.46	<0.01	25.59 ± 4.62	22.84 ± 2.55	<0.01
TC (mmol/L)	3.74 ± 1.22	4.08 ± 1.43	<0.01	4.57 ± 1.48	4.20 ± 1.37	<0.01
TG (mmol/L)	1.43 ± 0.84	1.06 ± 0.33	<0.001	2.15 ± 0.67	1.18 ± 0.52	<0.001
HDL-C (mmol/L)	1.12 ± 0.97	1.39 ± 0.71	<0.001	1.18 ± 0.42	1.46 ± 0.65	<0.001
LDL-C (mmol/L)	3.43 ± 1.66	2.76 ± 1.12	<0.001	3.27 ± 0.83	2.64 ± 1.34	<0.001
Cigarette smoking, no. (%)						
None	428 (47.66)	656 (71.69)	<0.001	381 (40.92)	690 (77.44)	<0.001
Smokers	470 (52.34)	259 (28.31)		550 (59.08)	201 (22.56)	
Triple-vessel disease, no. (%)	102 (11.36)	0 (0.00)	<0.001	97(10.42)	0 (0.00)	<0.001
ACS, no. (%)	509 (56.68)	0 (0.00)	<0.001	482 (51.77)	0 (0.00)	<0.001
PCI, no. (%)	477 (53.12)	0 (0.00)	<0.001	409 (43.93)	0 (0.00)	<0.001
CABG, no. (%)	113 (12.58)	0 (0.00)	<0.001	125 (13.43)	0 (0.00)	<0.001

Data are mean ± SD or no. (%) unless indicated. BMI: body mass index; TC: total cholesterol; TG: triglyceride; HDL-C: high-density lipoprotein cholesterol; LDL-C: low-density lipoprotein cholesterol; ACS, acute coronary syndrome; PCI, percutaneous coronary intervention; CABG, coronary artery bypass grafting.

**Table 2:** Genotype and allelic association analysis of polymorphisms in north and south China populations.

SNP ID	Genotype/allele	The northern China population				*p*^N#^	*p*^N*^	The southern China population				*p*^S#^	*p*^S*^
		Case no. (%)	Control no. (%)	OR^N^	95% CI^N^			Case no. (%)	Control no. (%)	OR^S^	95% CI^S^		
rs7216389	TT	416 (50.73)	408 (45.23)	1		0.011	0.022	517 (55.83)	461 (51.74)	1		0.013	0.026
(GSDMB)	CT	345 (42.07)	394 (43.68)	0.67	0.47–0.96			359 (38.77)	350 (39.28)	0.61	0.42–0.89		
	CC	59 (7.20)	100 (11.09)	0.58	0.41–0.82			50 (5.40)	80 (8.98)	0.56	0.38–0.81		
	T	1,177 (71.77)	1,210 (67.07)	1		0.003	0.006	1,393 (75.22)	1,272 (71.38)	1		0.009	0..018
	C	463 (28.23)	594 (32.93)	0.80	0.69–0.93			459 (24.78)	510 (28.62)	0.82	0.71–0.95		
rs9303277	CC	401 (45.52)	371 (42.16)	1		0.014	0.028	495 (56.96)	462 (53.47)	1		0.021	0.042
(IKZF3)	CT	405 (45.97)	396 (45.00)	0.65	0.47–0.90			326 (37.51)	325 (37.62)	0.62	0.42–0.92		
	TT	75 (8.51)	113 (12.84)	0.61	0.44–0.85			48 (5.52)	77 (8.91)	0.58	0.40–0.85		
	C	1,207 (68.50)	1,138 (64.66)	1		0.017	0.034	1,316 (75.72)	1,249 (72.28)	1		0.022	0.044
	T	555 (31.50)	622 (35.34)	0.84	0.73–0.97			422 (24.28)	479 (27.72)	0.84	0.72–0.97		

Data are no. (%) unless indicated.

^*^Corrected *p* value (after Bonferroni multiple adjustment).

^#^Adjusted by age, sex, BMI, serum lipid levels and tobacco smoking.

The N indicates the included northern Chinese Han population and S indicates the southern Chinese Han population.

SNP, single nucleotide polymorphism; OR, odds ratio; 95% CI, 95% confidence interval.
