# Supplementary material for: ORMDL3 contributes to the risk of atherosclerosis in Chinese Han population and mediates oxidized low-density lipoprotein-induced autophagy in endothelial cells
Source: Sci Rep. 2015 Nov 25;5:17194. doi: 10.1038/srep17194 (PMC4658630; doi:10.1038/srep17194)
Supplement: Supplementary Information [file srep17194-s1.pdf]

Supplementary Information

ORMDL3 contributes to the risk of atherosclerosis in Chinese Han population and mediates oxidized low-density lipoprotein-induced autophagy in endothelial cells

Xiaochun Ma, Rongfang Qiu, Jie Dang, Jiangxia Li, Qin Hu, Shan Shan, Qian Xin, Wenying Pan, XianliBian, Qianqian Yuan, Feng Long, Na Liu, Yan Li, Fei Gao, Chengwei Zou, Yaoqin Gong, Qiji Liu*

- **Supplementary Figure S1** ORMDL3 protein expression among the included subjects
- **Supplementary Figure S2** Ox-LDL up-regulates ORMDL3 expression in HAECs
- **Supplementary Figure S3** Autophagy is induced by ox-LDL in HAECs
- **Supplementary Figure S4** Knockdown of ORMDL3 confers no effect on protein levels of p-mTOR, total mTOR, p-AMPK and total AMPK in HUVECs
- **Supplementary Figure S5** Knockdown of ORMDL3 attenuates ox-LDL-induced TNF and IL-17A at the mRNA level in HUVECs

Supplementary figure legends

Supplementary Figure S1 ORMDL3 protein expression among the included subjects

Protein levels of ORMDL3 between AS cases and controls. The protein levels were normalized to that of ACTB.

Supplementary Figure S2 Ox-LDL up-regulates ORMDL3 in HAECs

(a) HAECs were incubated with ox-LDL at various concentrations (0, 50 and 100 μg/ml) for 12h. ORMDL3 protein expression was measured by immunoblot. (b) HAECs were stimulated with 100 μg/ml ox-LDL for the indicated durations (0, 4, 8 and 12h). ORMDL3 protein levels were examined by immunoblot.

Abbreviations: ox-LDL, oxidized low-density lipoprotein

Supplementary Figure S3 Autophagy is induced by ox-LDL in HAECs

(a) Following incubation with ox-LDL at various concentrations (0, 50 and 100 μg/ml) for 12h in HAECs, representative immunoblots of autophagy markers LC3 and SQSTM1/p62 were performed. (b) Immunoblot detection of protein levels of LC3 and SQSTM1/p62 in HAECs treated with 100 μg/ml ox-LDL for different durations (0, 4, 8 and 12h).

Abbreviations: ox-LDL, oxidized low-density lipoprotein; CQ, chloroquine

Supplementary Figure S4 Knockdown of ORMDL3 does not affect protein levels of p-mTOR, total mTOR, p-AMPK and total AMPK in HUVECs

At basal condition, in HUVECs stably transfected with ORMDL3 shRNA and control shRNA, protein levels of p-mTOR, total mTOR, p-AMPK and total AMPK wereevaluated by immunoblot.

Supplementary Figure S5 Knockdown of ORMDL3 attenuates ox-LDL-induced TNF and IL-17A expression at the mRNA level in HUVECs

The mRNA levels of TNF(a), IL-6 (b) and IL-17A (c) were determined by qRT-PCR in ShORMDL3 and shControl cells treated with 100 μg/ml ox-LDL or PBS for 12h.

Abbreviations: ox-LDL, oxidized low-density lipoprotein

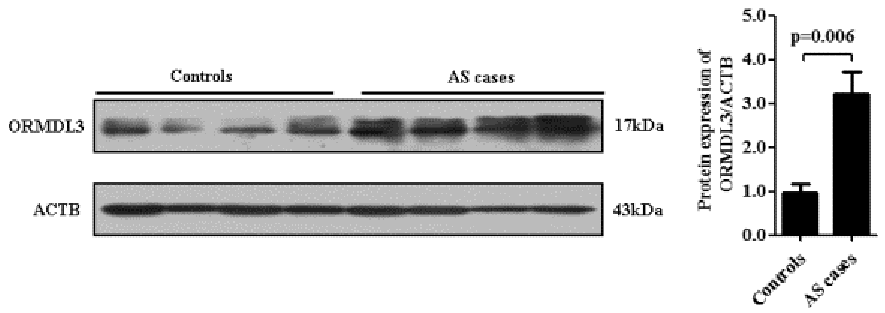

Supplementary Figure S1 ORMDL3 protein expression among the included subjects

Protein levels of ORMDL3 between AS cases and controls. The protein levels were normalized to that of ACTB.

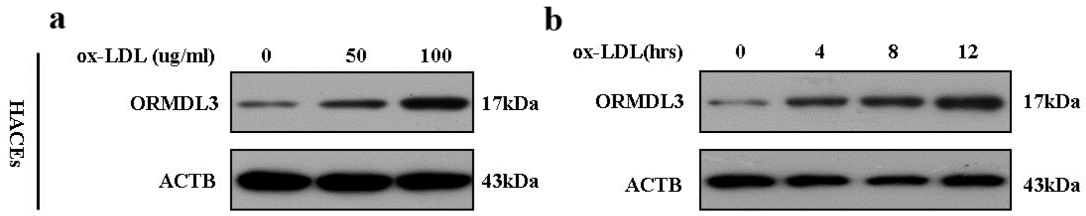

Supplementary Figure S2 Ox-LDL up-regulates ORMDL3 in HAECs

(a) HAECs were incubated with ox-LDL at various concentrations (0, 50 and 100 μg/ml) for 12h. ORMDL3 protein expression was measured by immunoblot. (b) HAECs were stimulated with 100 μg/ml ox-LDL for the indicated durations (0, 4, 8 and 12h). ORMDL3 protein levels were examined by immunoblot.

Abbreviations: ox-LDL, oxidized low-density lipoprotein

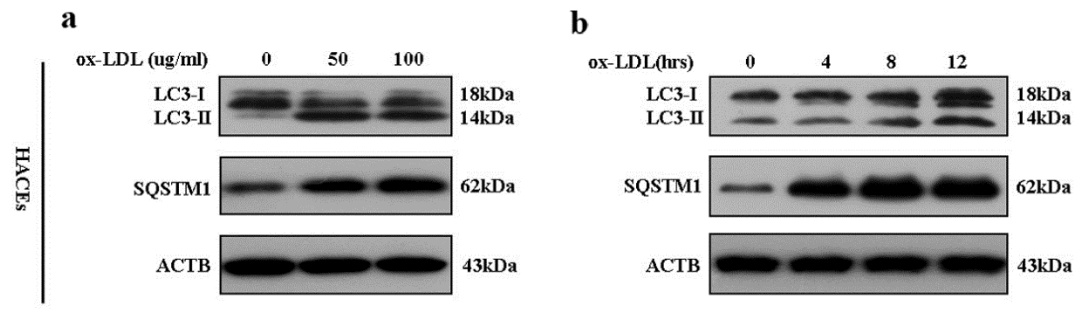

Supplementary Figure S3 Autophagy is induced by ox-LDL in HAECs

(a) Following incubation with ox-LDL at various concentrations (0, 50 and 100 μg/ml) for 12h in HAECs, representative immunoblots of autophagy markers LC3 and SQSTM1/p62 were performed. (b) Immunoblot detection of protein levels of LC3 and SQSTM1/p62 in HAECs treated with 100 μg/ml ox-LDL for different durations (0, 4, 8 and 12h). The protein levels of LC3 and SQSTM1/p62 were monitored by immunoblot.

Abbreviations: ox-LDL, oxidized low-density lipoprotein; CQ, chloroquine

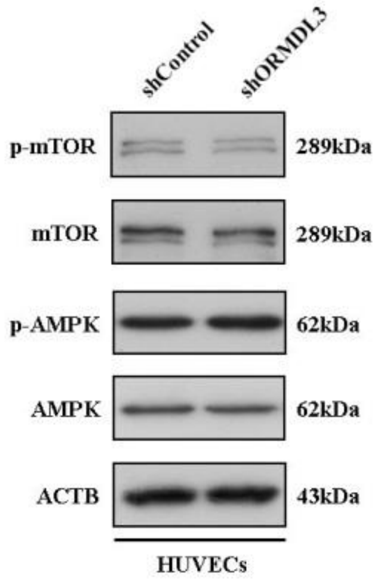

Supplementary Figure S4 Knockdown of ORMDL3 does not affect protein levels of p-mTOR, total mTOR, p-AMPK and total AMPK in HUVECs

At basal condition, in HUVECs stably transfected with ORMDL3 shRNA and control shRNA, protein levels of p-mTOR, total mTOR, p-AMPK and total AMPK wereevaluated by immunoblot.

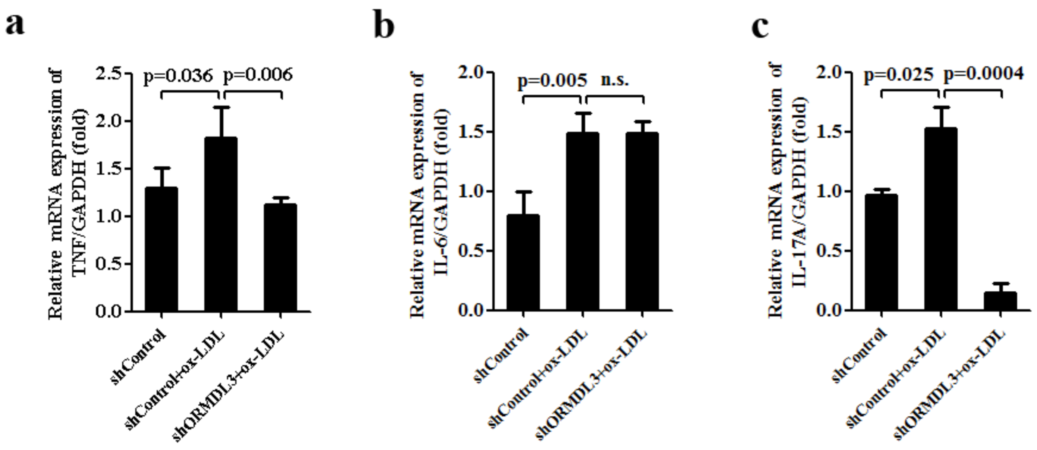

Supplementary Figure S5 Knockdown of ORMDL3 attenuates ox-LDL-induced TNF and IL-17A expression at the mRNA level in HUVECs

The mRNA levels of TNF(a), IL-6 (b) and IL-17A (c) were determined by qRT-PCR in ShORMDL3 and shControl cells treated with 100 μg/ml ox-LDL or PBS for 12h.

Abbreviations: ox-LDL, oxidized low-density lipoprotein
